# Supplementary material for: Preserved cognitive functions with age are determined by domain-dependent shifts in network responsivity
Source: Nat Commun. 2017 May 8;8:14743. doi: 10.1038/ncomms14743 (PMC5424147; doi:10.1038/ncomms14743)
Supplement: Supplementary Information — Supplementary Figures, Supplementary Tables, Supplementary Methods and Supplementary References [file ncomms14743-s1.pdf]

Supplementary Figures

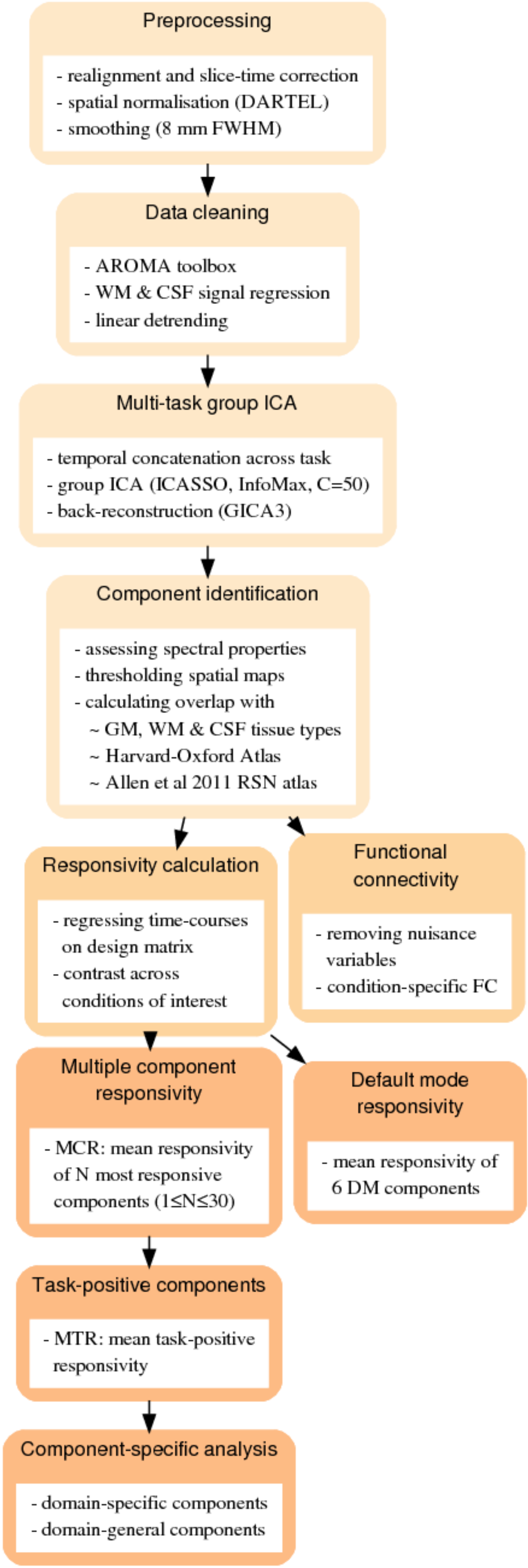

Supplementary Figure 1. Schematic of preprocessing pipeline. Major stages of preprocessing pipeline are grouped into separate boxes. See Methods for details and abbreviations.

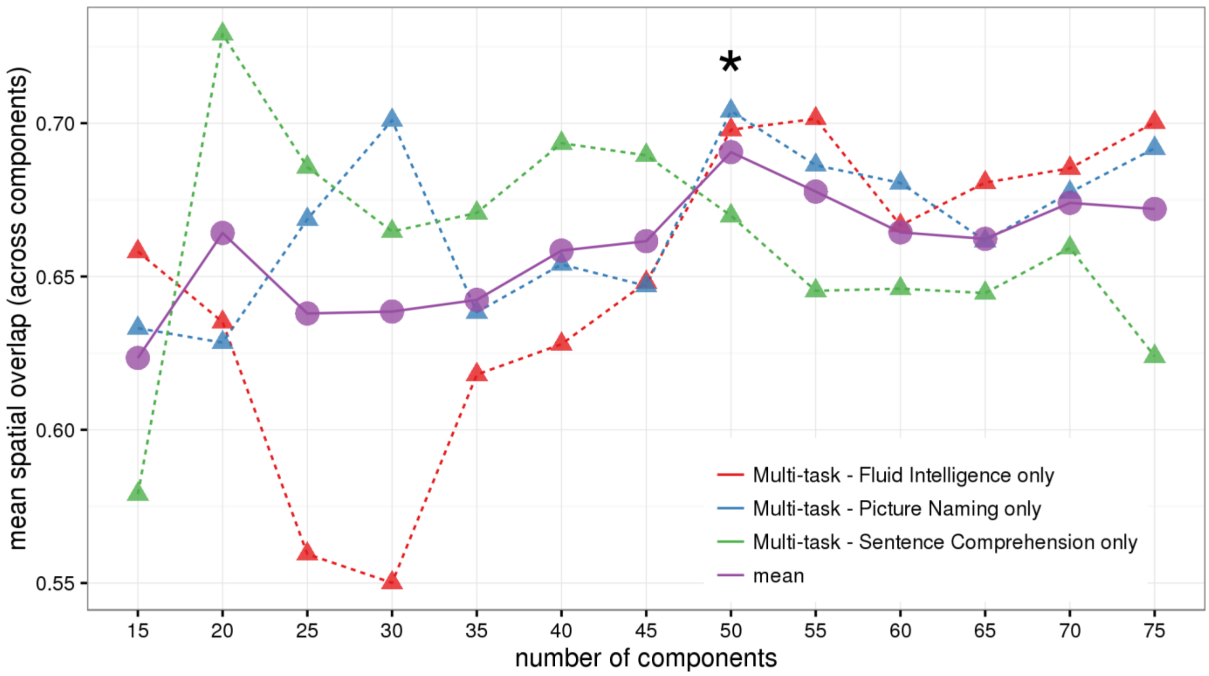

Supplementary Figure 2. Convergence test results between multiple-task versus single-task ICAs. We ran ICAs across a range of number of components (x axis) for each task individually (single -task ICA) and for temporally concatenated recordings all three tasks (multi -task ICA). In order to estimate spatial convergence between single-task and multi-task ICAs (y axis), we calculated the overlaps of the thresholded spatial maps after optimally pairing the components between the multi-task ICA and each single-task ICA results (see Supplementary Methods). Star at n = 50 denotes optimal convergence across tasks (maximal mean spatial overlap, purple line).

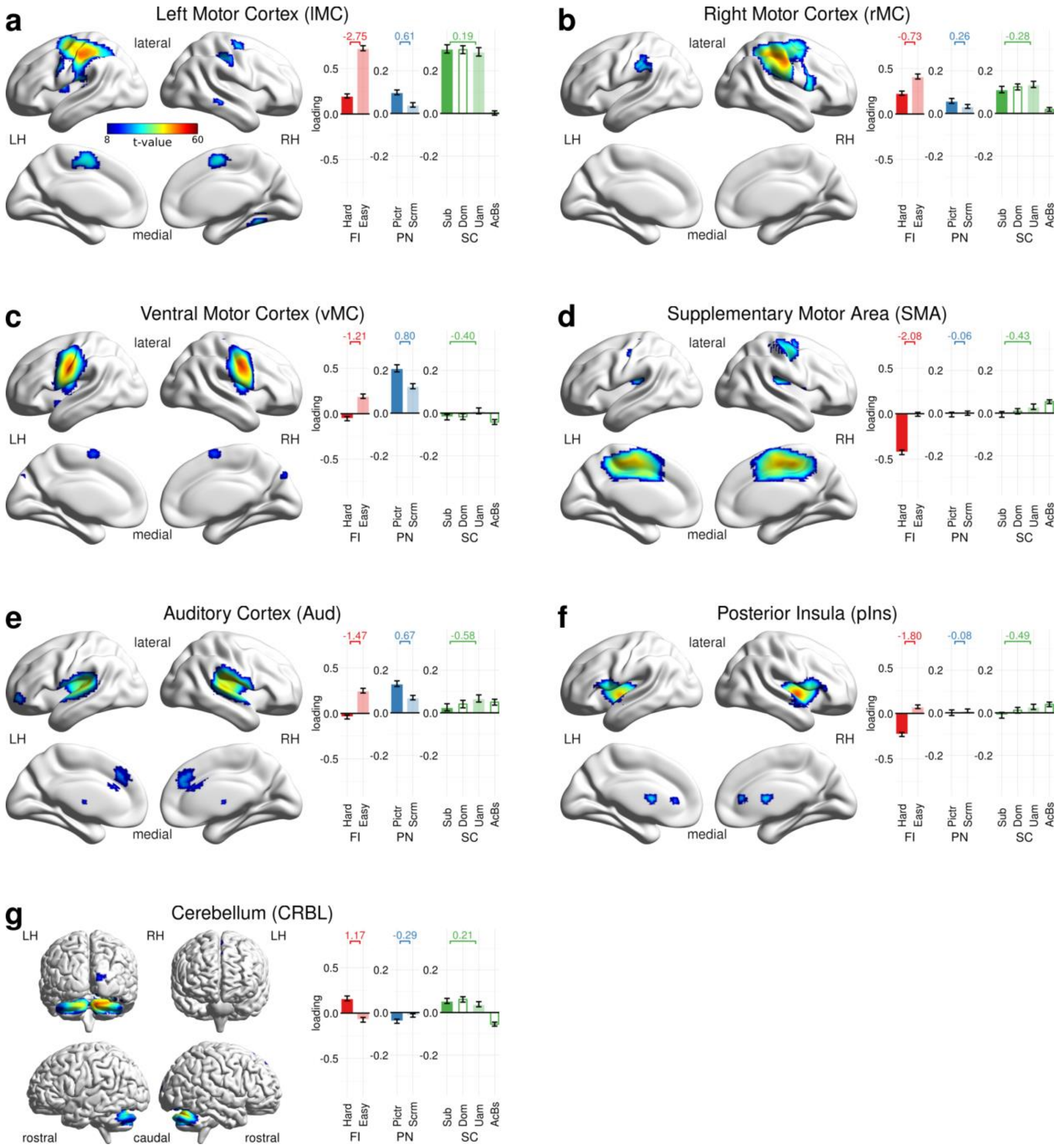

Supplementary Figure 3. Component spatial maps and loading values I: Motor, auditory, cerebellar and posterior insula components. Spatial maps (on the left) are mixture model thresholded t-maps at the group-level (see Methods), colour coded by group-level t-value from blue (low) to red (high). Bars and whiskers (on the right) denote cohort-mean loading values ± SEM, sorted by task (FI: Fluid Intelligence, PN: Picture Naming, SC: Sentence Comprehension), and higher (dark colour) versus lower (light colour) cognitive load (Hard: hard puzzle, Easy: easy puzzle, Pictr: picture naming, Scrm: scrambled image, Sub: subordinate sentences, Dom: dominant sentences, Uam: unambiguous sentences, AcBs: acoustic baseline, see Methods). The second and the fourth conditions of Sentence

Comprehension (Dom and AcBs) are not used in this study. Across-condition effect sizes (mean over SD of condition contrast) are given above the bars. LH/RH: left/right hemisphere.

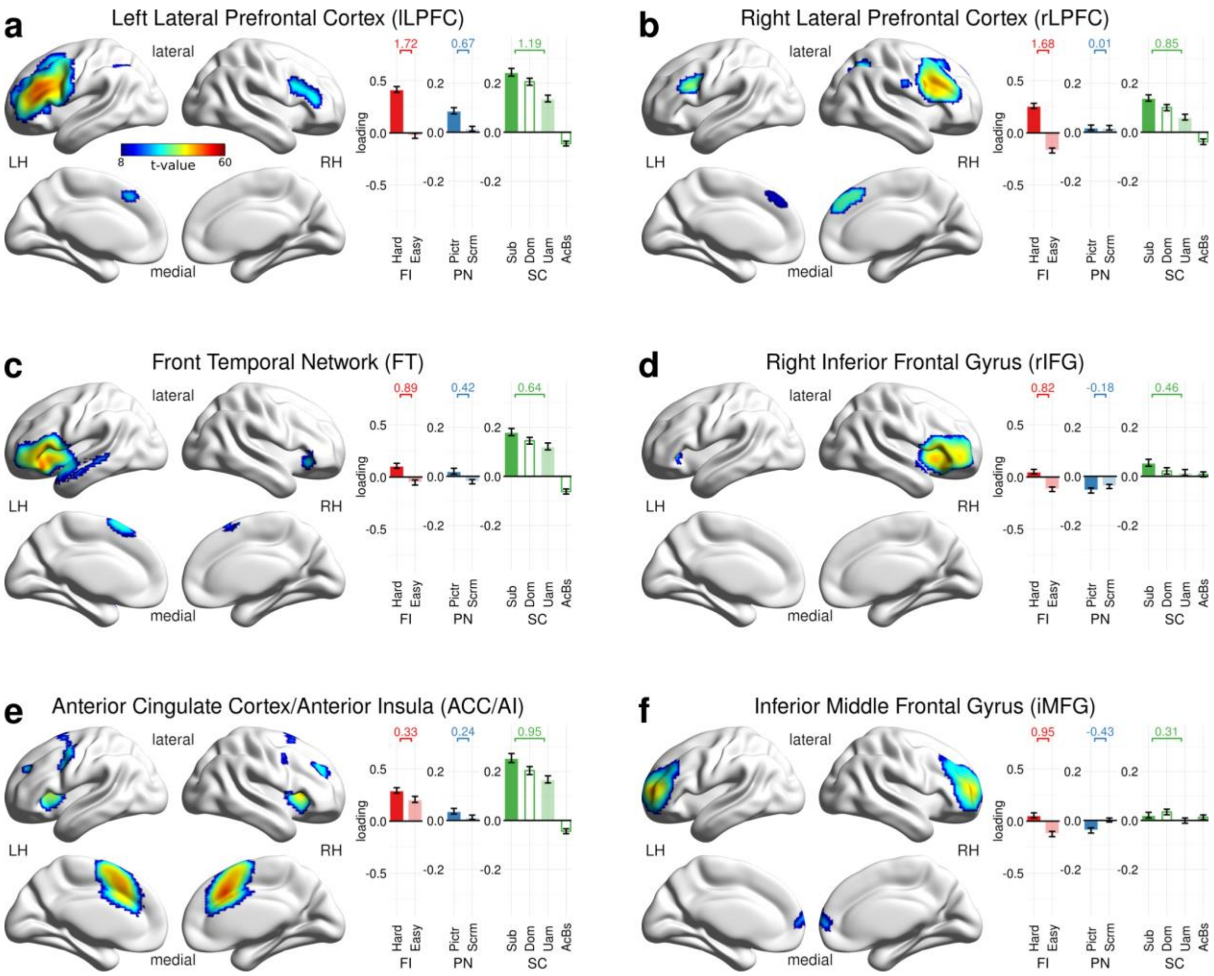

Supplementary Figure 4. Component spatial maps and loading values II: Frontal components. Spatial maps (on the left) are colour coded by group-level loading-value. Bars and whiskers (on the right) denote cohort-mean loading values ± SEM, sorted by task and higher (dark colour) versus lower (light colour) cognitive load. Across-condition effect sizes (mean over SD of condition contrast) are given above the bars. See Supplementary Fig. 3 for abbreviations and further details.

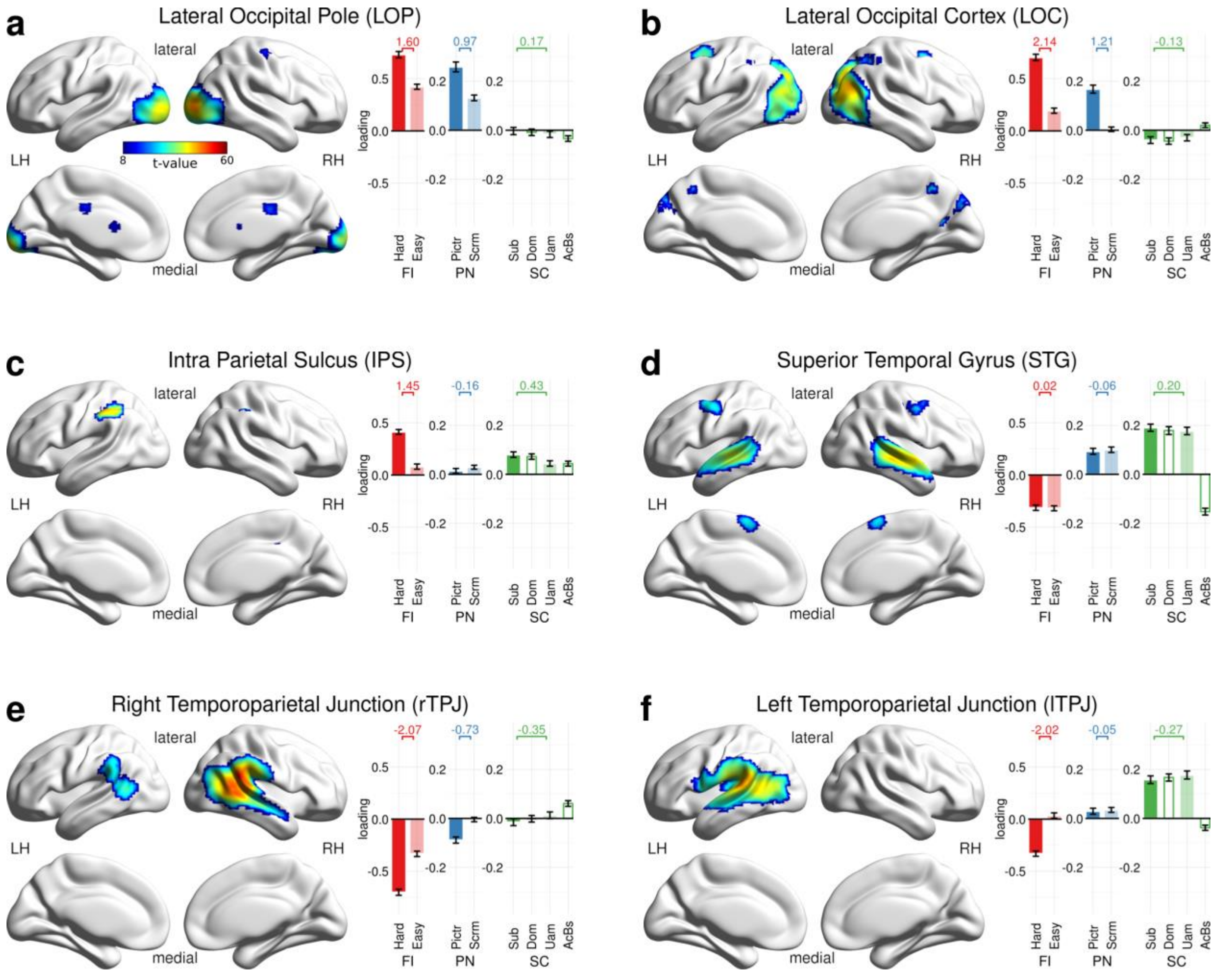

Supplementary Figure 5. Component spatial maps and loading values III: Dorsal visual and temporo-parietal components. Spatial maps (on the left) are colour coded by group-level loading-value. Bars and whiskers (on the right) denote cohort-mean loading values ± SEM, sorted by task and higher (dark colour) versus lower (light colour) cognitive load. Across-condition effect sizes (mean over SD of condition contrast) are given above the bars. See Supplementary Fig. 3 for abbreviations and further details.

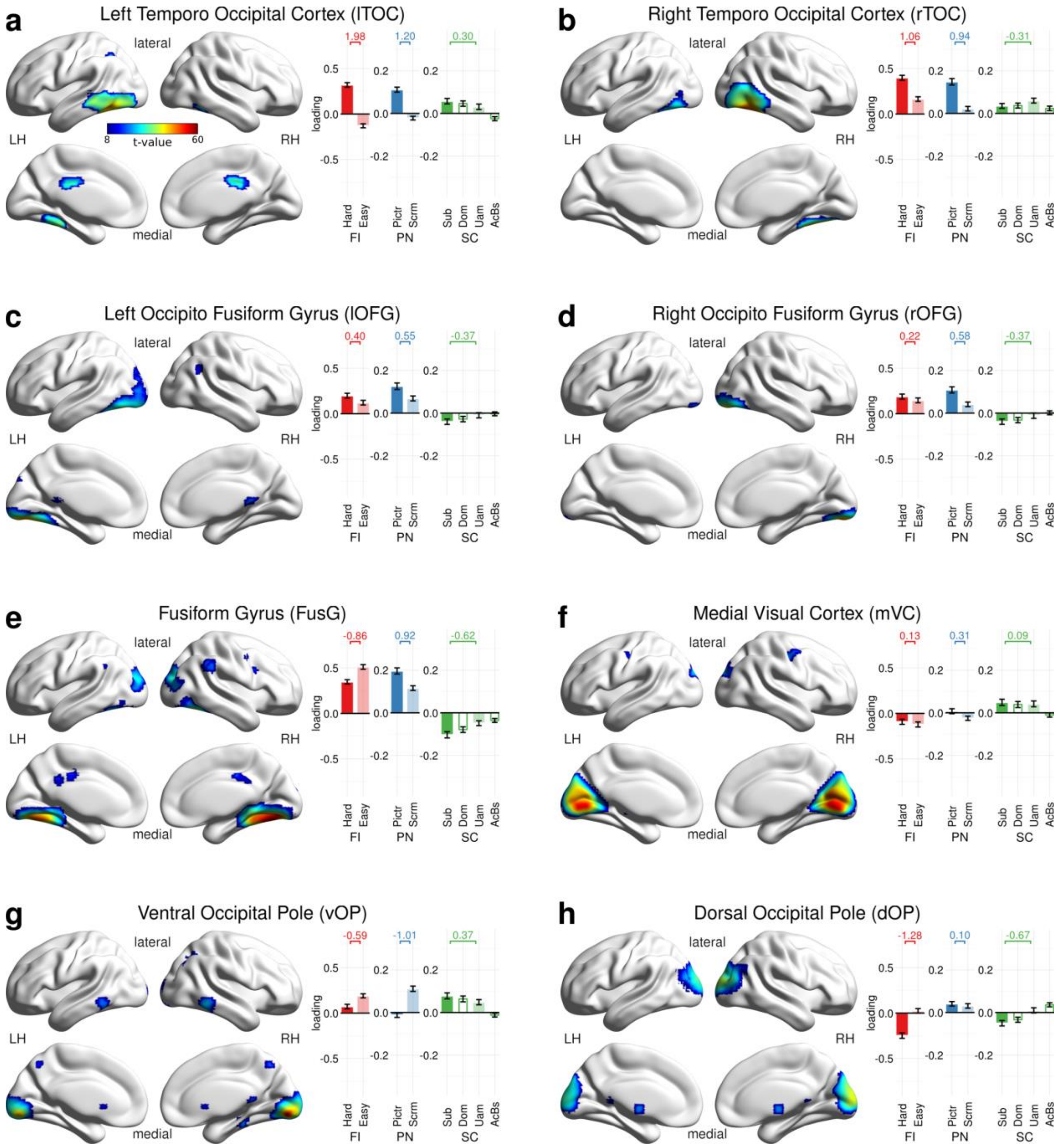

Supplementary Figure 6. Component spatial maps and loading values IV: Ventral visual components. Spatial maps (on the left) are colour coded by group-level loading-value. Bars and whiskers (on the right) denote cohort-mean loading values ± SEM, sorted by task and higher (dark colour) versus lower (light colour) cognitive load. Across-condition effect sizes (mean over SD of condition contrast) are given above the bars. See Supplementary Fig. 3 for abbreviations and further details.

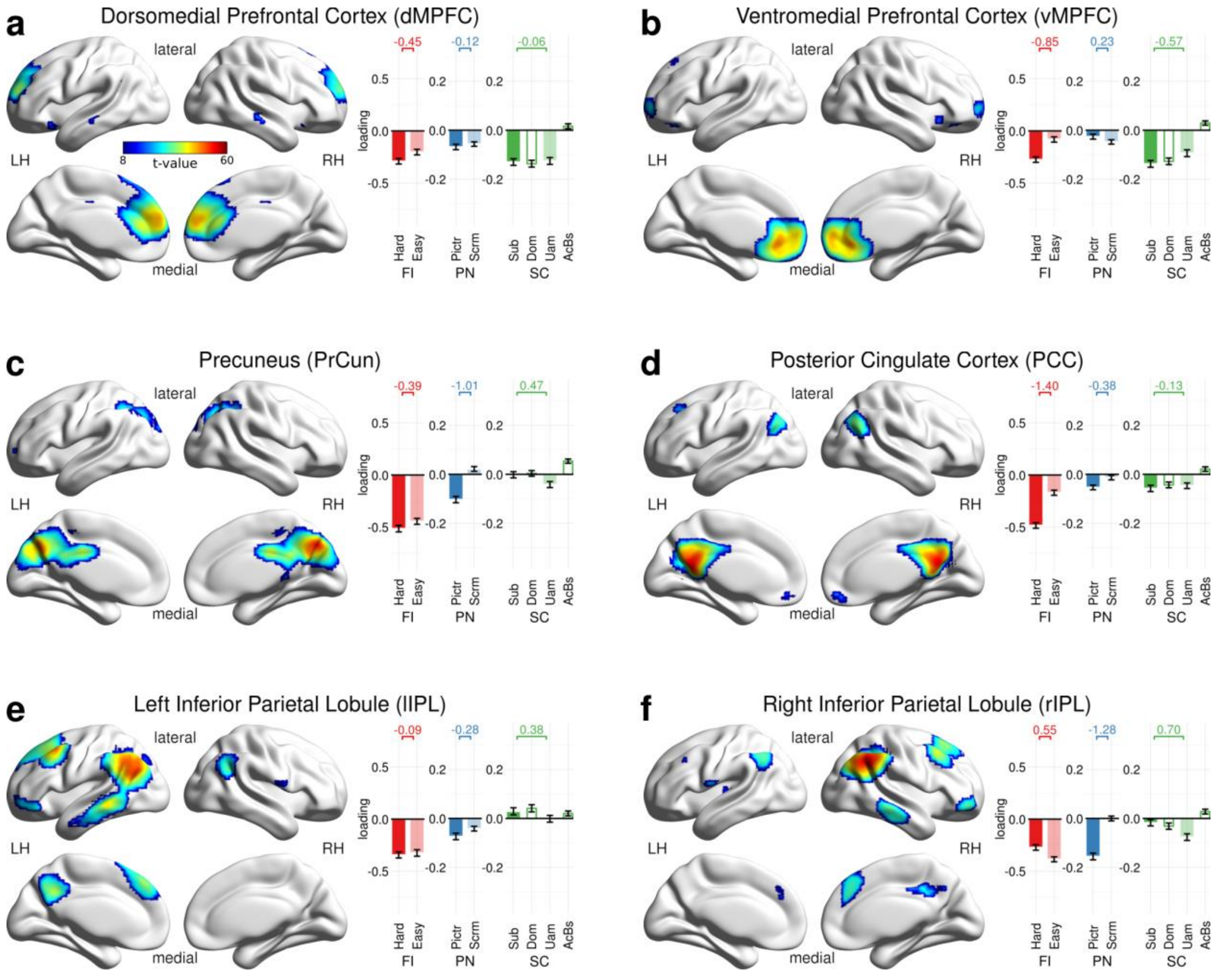

Supplementary Figure 7. Component spatial maps and loading values V: Default mode components. Spatial maps (on the left) are colour coded by group-level loading-value. Bars and whiskers (on the right) denote cohort-mean loading values ± SEM, sorted by task and higher (dark colour) versus lower (light colour) cognitive load. Across-condition effect sizes (mean over SD of condition contrast) are given above the bars. See Supplementary Fig. 3 for abbreviations and further details.

Supplementary Tables

Supplementary Table 1. Behavioural results.[i]

		Accuracy (%)				Reaction time (ms)			
		All participants	*Young*	*Middle-age*	*Older*	*All participants*	*Young*	*Middle-age*	*Older*
**Fluid Intelligence**	*Easy*	98.0 (0.3)	98.9 (0.2)	98.8 (0.2)	96.3 (0.7)	1067.5 (28.0)	939.9 (56.7)	1021.0 (40.1)	1224.2 (37.2)
	Hard	59.3 (2.1)	67.9 (3.3)	61.2 (3.5)	49.7 (3.3)	5281.1 (164.6)	5699.6 (267.6)	5243.3 (246.9)	4953.4 (325.9)
**Picture Naming**	*Pictures*	83.2 (0.8)	88.1 (0.8)	85.7 (1.1)	76.7 (1.2)	1024.1 (8.6)	1012.2 (16.4)	985.5 (9.8)	1073.1 (14.4)

i. Mean (and standard error of the mean) of accuracy/acceptability scores (percentage of correct/accepted responses) and reaction times (in milliseconds) are given for every scored condition (second column) of each tasks and for each age group.

		Acceptability (%)					Reaction time (ms)			
**Sentence Comprehension**	*Unambiguous*	93.2 (0.8)	94.9 (1.4)	95.3 (1.1)	89.7 (1.7)		1204.8 (40.1)	1030.9 (55.2)	1108.7 (51.6)	1452.1 (75.3)
	Dominant	84.8 (1.2)	88.9 (2.3)	86.6 (1.8)	79.5 (2.1)		1303.7 (39.5)	1135.8 (58.7)	1228.3 (55.6)	1524.5 (71.3)
	Subordinate	56.5 (2.3)	63.8 (4.6)	58.8 (3.9)	47.9 (3.2)		1545.7 (37.8)	1390.2 (61.8)	1465.5 (54.6)	1761.0 (62.8)

Supplementary Table 2. Component abbreviations, full names, categories (group), MNI coordinates and spatial overlap with two published functional brain atlases.[ii]

index	abbreviation	full name	group	MNI coordinate X	Y	Z	Allen et al, 2011 most overlapping IC	Allen et al, 2011 second most overlapping IC	Laird et al, 2011 most overlapping IC	Laird et al, 2011 second most overlapping IC
1	pIns	Posterior Insula	LMB	39	-3	0	IC21/BG 74%	IC17/STG-AUD 73%	ICN17/vMOT 51%	ICN16/STG-AUD 34%
2	Aud	Auditory Cortex	ASM	60	-18	12	IC17/STG-AUD 90%	IC7/vMot 45%	ICN16/STG-AUD 71%	ICN17/vMOT 41%
3	vMC	Ventral Motor Cortex	ASM	-60	-9	33	IC7/vMot 88%	IC56/preMOT 25%	ICN17/vMOT 82%	ICN5/Midbrain 31%
4	lMC	Left Motor Cortex	ASM	-51	-21	48	IC23/ldMot 81%	IC56/preMOT 37%	ICN8/vMOT 78%	ICN17/vMOT 33%
5	rMC	Right Motor	ASM	51	-30	48	IC24/rdMOT	IC38/pCG 53%	ICN17/vMOT	ICN8/vMOT 40%

ii. The table includes all 33 components of neural origin, three of which (motor components vMC, lMC and rMC) were removed from further analysis (see Methods in main text). Category abbreviations: LMB: limbic, ASM: auditory and somatomotor, ECC: executive and cognitive control, VIS: visual, CRL: cerebellum, DM: default mode. X, Y and Z give the MNI coordinates of the maximal value of the group level t-map for each component. The first and second most overlapping components, along with their their degree of overlap in percentage, are given for two published ICA atlases, a resting state atlas based on a large ageing population[1] and a task-based atlas combining recordings from thousands of experiments in a meta-analysis[2]. See original papers[1,2] for more information on these atlases and their components.

		Cortex					83%		52%	
6	SMA	Supplementary Motor Area	ASM	0	-12	48	IC29/supMOT 80%	IC56/preMOT 60%	ICN8/vMOT 77%	ICN13/DMN 41%
7	lTPJ	Left Temporoparietal Junction	ECC	-57	-30	15	IC17/STG-AUD 58%	IC71/pSTG 58%	ICN16/STG-AUD 47%	ICN17/vMOT 30%
8	rTPJ	Right Temporoparietal Junction	ECC	63	-45	21	IC71/pSTG 84%	IC17/STG-AUD 43%	ICN10/MTG-ITG 54%	ICN16/STG-AUD 30%
9	STG	Superior Temporal Gyrus	ECC	63	-24	-6	IC17/STG-AUD 79%	IC71/pSTG 51%	ICN16/STG-AUD 82%	ICN10/MTG-ITG 14%
10	ACC/AI	Anterior Cingulate Cortex/Anterior Insula	ECC	6	21	36	IC55/ACC-AI 76%	IC56/preMOT 61%	ICN6/SMA-FEF 57%	ICN20/Artef 42%
11	iMFG	Inferior Middle Frontal Gyrus	ECC	-30	57	9	IC49/rMFG 52%	IC68/MFG 41%	ICN15/rFP 26%	ICN2/OFC 16%
12	rIFG	Right Inferior Frontal Gyrus	ECC	48	21	0	IC42/rIFG 89%	IC21/BG 50%	ICN15/rFP 53%	ICN4/ACC-AI 40%
13	FT	Front Temporal Network	ECC	-45	24	-6	IC20/lIFG 70%	IC55/ACC-AI 46%	ICN18/lFP 49%	ICN4/ACC-AI 43%

14	lLPFC	Left Lateral Prefrontal Cortex	ECC	-48	36	15	IC47/MFG 64%	IC20/lIFG 38%	ICN18/lFP 70%	ICN17/vMOT 15%
15	rLPFC	Right Lateral Prefrontal Cortex	ECC	54	12	30	IC60/rIPL-rMFG 81%	IC47/MFG 81%	ICN15/rFP 46%	ICN20/Artef 28%
16	IPS	Intra Parietal Sulcus	ECC	-54	-36	45	IC38/pCG 99%	IC34/lIPL-lMFG 90%	ICN7/MFG-SPL 53%	ICN17/vMOT 46%
17	LOC	Lateral Occipital Cortex	VIS	36	-78	24	IC52/AngG 55%	IC59/Cuneus 55%	ICN7/MFG-SPL 38%	ICN10/MTG-ITG 35%
18	lTOC	Left Temporo Occipital Cortex	VIS	-48	-57	-15	IC39/pITG 55%	IC52/AngG 41%	ICN18/lFP 41%	ICN10/MTG-ITG 40%
19	rTOC	Right Temporo Occipital Cortex	VIS	42	-66	-18	IC39/pITG 88%	IC48/lLingG 50%	ICN10/MTG-ITG 54%	ICN11/lpOcc 54%
20	lOFG	Left Occipito Fusiform Gyrus	VIS	-30	-78	-18	IC48/lLingG 55%	IC67/mLingG 47%	ICN14/CRBL 69%	ICN11/lpOcc 54%
21	rOFG	Right Occipito Fusiform Gyrus	VIS	27	-84	-12	IC48/lLingG 65%	IC67/mLingG 63%	ICN11/lpOcc 75%	ICN14/CRBL 56%
22	FusG	Fusiform Gyrus	VIS	30	-63	-15	IC67/mLingG 83%	IC48/lLingG 65%	ICN14/CRBL 54%	ICN12/mpOCC 43%
23	mVC	Medial Visual	VIS	0	-78	0	IC64/CalcG 95%	IC46/pLingG 59%	ICN12/mpOCC	ICN9/SPL 28%

		Cortex							98%	
24	vOP	Ventral Occipital Pole	VIS	6	-84	-9	IC46/pLingG 85%	IC67/mLingG 61%	ICN12/mpOCC 68%	ICN11/lpOcc 54%
25	dOP	Dorsal Occipital Pole	VIS	3	-96	6	IC59/Cuneus 72%	IC46/pLingG 71%	ICN12/mpOCC 69%	ICN11/lpOcc 50%
26	LOP	Lateral Occipital Pole	VIS	30	-93	3	IC48/lLingG 70%	IC46/pLingG 55%	ICN11/lpOcc 90%	ICN12/mpOCC 30%
27	CRBL	Cerebellum	CRL	33	-75	-27	IC67/mLingG 55%	IC46/pLingG 36%	ICN14/CRBL 97%	ICN11/lpOcc 17%
28	lIPL	Left Inferior Parietal Lobule	DM	-45	-66	30	IC34/lIPL-lMFG 79%	IC53/PCC 37%	ICN13/DMN 24%	ICN18/lFP 21%
29	rIPL	Right Inferior Parietal Lobule	DM	54	-54	36	IC60/rIPL-rMFG 88%	IC34/lIPL-lMFG 43%	ICN7/MFG-SPL 28%	ICN13/DMN 27%
30	dMPFC	Dorsomedial Prefrontal Cortex	DM	3	48	18	IC25/vACC 63%	IC68/MFG 49%	ICN2/OFC 31%	ICN20/Artef 23%
31	vMPFC	Ventromedial Prefrontal Cortex	DM	0	54	-3	IC25/vACC 89%	IC55/ACC-AI 17%	ICN2/OFC 95%	ICN3/BG 4%
32	PrCun	Precuneus	DM	9	-72	36	IC50/PreC 90%	IC53/PCC 66%	ICN13/DMN 63%	ICN12/mpOCC 43%

33	PCC	Posterior Cingulate Cortex	DM	6	-54	21	IC53/PCC 99%	IC50/PreC 47%	ICN13/DMN 79%	ICN9/SPL 33%

Supplementary Table 3. Compensation analysis results.[iii]

	Component	Test 1: Age-related change in component responsivity			Test 2: Moderation by age of the relation between component responsivity and task score		
		Fluid Intelligence	Picture Naming	Syntactic Comprehension	Fluid Intelligence	Picture Naming	Syntactic Comprehension
1	pIns	0.32 (0.00*)	-0.07 (0.51)	0.29 (0.00*)	-0.50 (0.20)	-0.04 (0.96)	1.95 (0.14)
2	Aud	0.30 (0.01*)	-0.36 (0.00*)	0.18 (0.09)	-0.43 (0.24)	1.17 (0.17)	1.11 (0.47)
3	SMA	0.49 (0.00*)	0.16 (0.14)	0.19 (0.08)	-0.45 (0.20)	-1.34 (0.17)	-0.07 (0.96)
4	lTPJ	0.07 (0.49)	0.03 (0.77)	0.31 (0.00*)	-0.49 (0.20)	0.42 (0.64)	1.07 (0.48)
5	rTPJ	0.23 (0.03)	0.20 (0.04)	0.24 (0.02)	-0.87 (0.02)	-0.11 (0.89)	1.59 (0.23)
6	STG	0.25 (0.02)	0.02 (0.86)	0.00 (0.98)	-0.49 (0.19)	-0.87 (0.22)	-0.91 (0.54)
7	ACC/AI	-0.33 (0.00*)	-0.14 (0.17)	0.01 (0.94)	0.20 (0.47)	-0.99 (0.27)	1.36 (0.17)

iii. Two tests were used to detect potential age-related compensation effects in each component (rows) and task (columns). Test 1, age-related change in component responsivity, reveals a potential effect of compensation if a component with positive responsivity is significantly positively correlated to age. Test 2, age-related change in the relation between component responsivity and task score, reveals an effect of compensation if age significantly and positively moderates the relation between responsivity and task score (see also Results in main text). Values given are standardized regression coefficients of age (Test 1) and age x responsivity interaction (Test 2), and their p-values (in parentheses) with '*' added for $p < 0.01$. Note that while some of the components show significant age-related changes in their responsivity in Test 1, none of these cases satisfies both criteria of a potential compensation effect (that is, positive mean responsivity and positive correlation to age). We also note that the selected p-threshold, uncorrected for multiple comparisons, is rather lenient considering the number of tests performed, strengthening the confidence in these negative results. For full names of components, see Supplementary Table 2.

8	iMFG	-0.22 (0.03)	0.05 (0.64)	0.00 (0.98)	0.28 (0.50)	0.03 (0.97)	1.77 (0.18)
9	rIFG	-0.21 (0.04)	0.01 (0.90)	-0.09 (0.37)	0.38 (0.33)	-1.61 (0.08)	0.53 (0.64)
10	FT	-0.18 (0.10)	-0.02 (0.86)	-0.02 (0.84)	0.40 (0.33)	-0.22 (0.77)	-1.01 (0.40)
11	lLPFC	-0.64 (0.00*)	-0.41 (0.00*)	-0.14 (0.18)	0.09 (0.78)	-0.95 (0.26)	2.00 (0.06)
12	rLPFC	-0.60 (0.00*)	-0.21 (0.04)	-0.16 (0.10)	0.45 (0.12)	0.10 (0.90)	1.47 (0.17)
13	IPS	-0.50 (0.00*)	-0.15 (0.16)	0.00 (0.97)	0.25 (0.42)	-2.07 (0.02)	1.62 (0.19)
14	LOC	-0.68 (0.00*)	-0.41 (0.00*)	0.23 (0.03)	0.13 (0.70)	-0.65 (0.38)	0.65 (0.67)
15	lTOC	-0.55 (0.00*)	-0.25 (0.01)	-0.21 (0.04)	0.09 (0.78)	-0.70 (0.35)	0.09 (0.94)
16	rTOC	-0.51 (0.00*)	-0.28 (0.01*)	0.19 (0.07)	0.16 (0.64)	-0.30 (0.63)	0.18 (0.88)
17	lOFG	-0.25 (0.02)	-0.17 (0.10)	0.18 (0.09)	-0.07 (0.86)	0.42 (0.63)	1.36 (0.26)
18	rOFG	-0.11 (0.29)	-0.15 (0.16)	0.03 (0.79)	-0.21 (0.60)	-0.48 (0.46)	0.72 (0.60)
19	FusG	-0.16 (0.14)	-0.20 (0.06)	0.19 (0.07)	-0.83 (0.02)	0.51 (0.60)	0.06 (0.96)
20	mVC	-0.17 (0.11)	-0.04 (0.70)	0.12 (0.26)	0.13 (0.71)	-0.07 (0.93)	1.02 (0.39)
21	vOP	-0.09 (0.42)	0.41 (0.00*)	-0.07 (0.50)	0.24 (0.43)	-0.31 (0.66)	1.02 (0.41)
22	dOP	0.40 (0.00*)	0.11 (0.29)	0.15 (0.15)	-0.14 (0.67)	0.01 (0.99)	-1.20 (0.25)
23	LOP	-0.48 (0.00*)	-0.33 (0.00*)	-0.02 (0.86)	0.13 (0.73)	0.57 (0.40)	2.78 (0.03)
24	CRBL	-0.13 (0.21)	-0.01 (0.90)	0.11 (0.30)	-0.08 (0.82)	-1.02 (0.23)	-0.69 (0.60)

25	lIPL	0.41 (0.00*)	0.20 (0.05)	-0.04 (0.73)	-0.26 (0.47)	-1.29 (0.06)	-1.35 (0.32)
26	rIPL	0.19 (0.08)	0.35 (0.00*)	-0.10 (0.35)	0.86 (0.01)	-0.45 (0.52)	-0.57 (0.63)
27	dMPFC	0.41 (0.00*)	0.20 (0.06)	0.07 (0.52)	-0.22 (0.59)	-2.05 (0.02)	-0.68 (0.65)
28	vMPFC	0.51 (0.00*)	0.09 (0.40)	-0.22 (0.04)	-0.33 (0.28)	1.01 (0.23)	-1.39 (0.27)
29	PrCun	0.01 (0.90)	0.41 (0.00*)	-0.15 (0.15)	0.08 (0.82)	-0.54 (0.44)	1.21 (0.28)
30	PCC	0.48 (0.00*)	0.21 (0.05)	-0.01 (0.96)	-0.42 (0.18)	-0.36 (0.64)	-2.02 (0.09)

Supplementary Methods

Selecting ICA resolution

In order to determine the optimal number of components of the multi-task ICA (see Methods), we ran ICAs at a range of resolutions (between 15 and 75 components, by an increment of 5) for each individual task (single-task ICAs) as well as using data from all three tasks (multi-task ICA, Supplementary Fig. 2). At each resolution, we estimated the convergence between the obtained component sets of the multi-task and single-task ICAs. More specifically, for each single-task ICA, we calculated the mean spatial overlap across an optimal pairing of single-task – multi-task components. We found this optimal component pairing between ICA runs using the so-called Hungarian algorithm, a linear programming technique that maximises the mean of pair-wise overlaps for all the pairs across the two component sets by solving the analogous linear sum assignment problem.

We found optimal convergence between multiple-task and single-task ICA components at the resolution of n = 50 components (mean spatial overlap across components and tasks: 69%, Supplementary Fig. 2). Additionally, this resolution also had one of the lowest spread of component-averaged spatial similarity values across tasks (Fluid Intelligence: 69%, Picture Naming: 70%, Sentence Comprehension: 67%), ensuring that no task dominated over the others in the final common functional components (see also supplementary section Single-task ICA results). We note that the average spatial similarity value between single- and multiple-task ICA components increased further (to 71%) after excluding the 17 non-neuronal components from the calculation (see Methods). We also note that the algorithm we used to find the optimal component pairings across ICA decompositions was not designed to find correspondence between potential split or merged components (a situation conceivable across ICA runs), and therefore the reported overlap values may represent an underestimation of a possible alternative metric that does account for such splits and merges between component sets. Nevertheless, based on our robustness tests at a lower (n = 20) and a higher (n = 70) resolution ICA decomposition, we report that the specific number of ICA components chosen did not significantly affect the main results of our analysis.

Modelling of fMRI recordings by task conditions and events

In this section, we first discuss how component responsivities were calculated in general, and then give details on how each task was modelled by its specific experimental conditions.

For each of the three tasks, component responsivity (modulation of component activity by the task) was calculated as follows. First, for each component and subject, the subject-specific component time-course was regressed in a multiple linear regression on the task's design matrix. The design matrix consisted of haemodynamic response function (HRF) convolved time-courses of all the experimentally manipulated task conditions and events (see task-specific descriptions below for details), the 6 standard realignment parameters (accounting for in-scanner head-motion) and a regression constant. This procedure is formally the same as the usual regression step of the design matrix on the voxel's time-course during the generation of a standard SPM activation map, but done much fewer times here (once for each of the 30 components) than in a voxel-wise SPM analysis (for tens of thousands of voxels), thereby greatly easing SPM's massive multiple comparisons problem[3].

The result of this step was a standardised β loading value for each experimental condition on each component and for each subject, representing the extent to which that component was modulated (activated or de-activated) by the corresponding conditions of interest during the task for a given subject. Based on these loading values and the conditions of interest for each task (see Methods), subject-specific contrast values were calculated for each component as the difference between the β loadings of the conditions of interest. Taking into account the nature of the conditions, these contrast or responsivity values were intended to represent the amount of excess activation/suppression of the component from the less demanding condition to the more demanding one for each subject. Below, we give details on how each task was modelled by its specific experimental conditions.

Fluid Intelligence. For this experiment we used the standardised Cattell Culture Fair test of fluid intelligence, modified for use in the scanner (see Methods). For the modelling of this self-paced, block-design task, the two alternating difficulty levels, easy and hard puzzles, provided the conditions of interest. Each block lasted 30 seconds, with four blocks at each difficulty level. We used the contrast between the loading values of the two levels to estimate neural responsivity to fluid intelligence processes.

Picture Naming. This task measures word retrieval during naming pictures of objects with common names (see Methods). The model of the task comprised of the three task conditions: fixation point, scrambled image and object image. Onset of each trial was defined as the onset of appearance on display, with duration up to subject response (extracted from the recorded auditory responses). Object image trials were further categorised into correctly and incorrectly named trials, in order to enhance the contrast between naming and the baseline conditions. We used the contrast between the correctly named and scrambled conditions to estimate neural responsivity to object recognition and lexical retrieval processes.

Sentence Comprehension. This experiment investigates syntactic processing using syntactically ambiguous sentences (see Methods). In order to maximize sensitivity to the processing of syntactic ambiguity, we used an event model that separates the period immediately following the ambiguous phrase. To this end, the onset of each condition was defined separately as the onset of the disambiguating verb following the (ambiguous or unambiguous) phrase, with a variable duration equivalent to the remaining length of the phrase (mean duration: 403 ± 30 ms). We also included an extra regressor of no interest with onset at the start of each sentence and duration up to the onset of the disambiguating verb (mean duration: 2185 ± 317 ms). This model tests for effects of the second half of the sentence (the disambiguating verb) while controlling for those of the first half (ambiguous/unambiguous phrase). In addition to the first-half of the sentence, four task conditions

were modelled, corresponding to the different combinations of sentence and continuation word types: 1) subordinate (unexpected) continuations to ambiguous sentences, 2) dominant (highly predicted) continuations to ambiguous sentences, 3) matched continuations to syntactically unambiguous sentences, and 4) an acoustic baseline condition (musical rain). We used the contrast between subordinate and unambiguous conditions to estimate responsivity to syntactic processing demands.

Robustness test for MCR

A potential explanation for the observed increase in prediction power from single component to multiple component (task-positive) responsivity is that MCR is simply less noisy than the single responsivity values of individual components, and although all four task-positive components are important for cognition, in fact they do not each carry independent contribution to performance. We tested this possibility by running a series of multiple linear regressions (instead of correlation tests used in the main text) and calculating the proportion of variance in task score explained by each component set, adjusted for the number of components in the model (adjusted R-squared). The results were identical to the simpler averaging approach used in the main text (see Methods), with the n = 4 most highly responsive components yielding the highest adjusted R-squared value for each task. This indicates that the first four most responsive components do indeed contribute independently to performance, while there is no evidence for such independent contribution from additional components. Nonetheless, we note that our main findings are fairly robust against changing the number of components comprising the task-positive set, yielding qualitatively the same, although quantitatively progressively weakening, results up to n = 10 components.

Single-task ICA results

Our choice for running a joint ICA on multiple tasks (multi-task ICA) was motivated by our aim to compare the (domain-specific and domain-general) functional brain components across cognitive domains. This method, however, may inadvertently bias some of the components of some task(s) by those of other tasks (see explanation in Methods). To minimise this effect, we took the precaution to carefully select the optimal number of components (n=50) estimated by the largest convergence between single- and multiple-task components (see Methods in main text and supplementary section Selecting ICA resolution). In addition, we also tested the robustness of the results of the multiple-task ICA against those of standard single-task ICA analysis.

In line with our results on the convergence of components between single- and multi-task ICAs (see Methods), we found highly similar sets of components across all tasks (average spatial overlap between optimal component pairing of single- and multiple-task ICAs: Fluid Intelligence: 68%, Picture Naming: 71%, Sentence Comprehension: 74%, after excluding noise/vascular components). The only notable exceptions are two task-positive components of Fluid Intelligence, the left and right LPFCs, which were merged into a single component by the single-task ICA on Fluid Intelligence (but were left separated in the single-task ICAs of the other two tasks). This difference can be explained by the stronger and more accordant responsivity, and therefore tighter interconnectivity, of these task-positive components in Fluid Intelligence than in the other two tasks. Importantly, however, the result that these components were recruited to a largely disparate extent in the other two tasks (see Supplementary Fig. 4) suggests that they are in fact separable functional components (at least in some cognitive contexts), highlighting the strength of the multi-task ICA approach in obtaining a canonical, task-independent set of functional brain components for comparative multiple-task analysis.

Nevertheless, analysis of the components obtained through single-task ICAs qualitatively replicated our main findings. Specifically, in both declining tasks, MTRs of single-task ICAs showed strong age-related decreases with age and significantly mediated the age-related cognitive difference. Furthermore, MTR did not show any age-related difference for the cognitively preserved Sentence Comprehension. Beyond these correspondence between results on single-task and multi-

task ICAs, we also report a slight general increase in strength of some of the effects in the single-task analysis relative to the multi-task analysis. This slight increase in effect size, despite ability of MTR to account for merging and splitting of some components, may reflect the presence of subtle task-dependent differences in the spatial configuration of the functional components, pointing to the importance of characterising these potential differences and their contribution to cognition in future higher-resolution studies. Nevertheless, we emphasize that, in assessing their task-dependent functional responsivity, the current study utilised the correspondence between the tasks' brain components for across-tasks comparisons, which feature is to a large extent lost if the component sets are obtained from different single-task ICAs.

Supplementary References

1. Allen, E. A. *et al.* A Baseline for the Multivariate Comparison of Resting-State Networks. *Front. Syst. Neurosci.* **5,** (2011).
2. Laird, A. R. *et al.* Behavioral interpretations of intrinsic connectivity networks. *J. Cogn. Neurosci.* **23,** 4022–4037 (2011).
3. Eklund, A., Nichols, T. E. & Knutsson, H. Cluster failure: why fMRI inferences for spatial extent have inflated false-positive rates. *Proc. Natl. Acad. Sci.* 201602413 (2016).
